# MER: a shell script and annotation server for minimal named entity recognition and linking

**DOI:** 10.1186/s13321-018-0312-9

**Published:** 2018-12-05

**Authors:** Francisco M. Couto, Andre Lamurias

**Affiliations:** 1 LASIGE, Faculdade de Ciências, Universidade de Lisboa, 1749 016 Lisbon, Portugal; 2 Faculty of Sciences, BioISI - Biosystems and Integrative Sciences Institute, University of Lisboa, Campo Grande, C8 bdg, 1749 016 Lisbon, Portugal

**Keywords:** Named-entity recognition, Entity linking, Annotation server, Text mining, Biomedical ontologies, Lexicon

## Abstract

Named-entity recognition aims at identifying the fragments of text that mention entities of interest, that afterwards could be linked to a knowledge base where those entities are described. This manuscript presents our minimal named-entity recognition and linking tool (MER), designed with flexibility, autonomy and efficiency in mind. To annotate a given text, MER only requires: (1) a lexicon (text file) with the list of terms representing the entities of interest; (2) optionally a tab-separated values file with a link for each term; (3) and a Unix shell. Alternatively, the user can provide an ontology from where MER will automatically generate the lexicon and links files. The efficiency of MER derives from exploring the high performance and reliability of the text processing command-line tools grep and awk, and a novel inverted recognition technique. MER was deployed in a cloud infrastructure using multiple Virtual Machines to work as an annotation server and participate in the Technical Interoperability and Performance of annotation Servers task of BioCreative V.5. The results show that our solution processed each document (text retrieval and annotation) in less than 3 s on average without using any type of cache. MER was also compared to a state-of-the-art dictionary lookup solution obtaining competitive results not only in computational performance but also in precision and recall. MER is publicly available in a GitHub repository (https://github.com/lasigeBioTM/MER) and through a RESTful Web service (http://labs.fc.ul.pt/mer/).

## Introduction

Text has been and continues to be for humans the traditional and natural mean of representing and sharing knowledge. However, the information encoded in free text is not easily attainable by computer applications. Usually, the first step to untangle this information is to perform named-entity recognition (NER), a text mining task for identifying mentions of entities in a given text [1–3]. The second step is linking these mentions to the most appropriate entry in a knowledge base. This last step is usually referred to as the named-entity linking (NEL) task but is also referred to as entity disambiguation, resolution, mapping, matching or even grounding [4].

State-of-the-art NER and NEL solutions are mostly based on machine learning techniques, such as Conditional Random Fields and/or Deep Learning [5–14]. These solutions usually require as input a training corpus, which consists of a set of texts and the entities mentioned on them, including their exact location (annotations), and the entries in a knowledge base that represent these entities [15]. The training corpus is used to generate a model, which will then be used to recognize and link entities in new texts. Their effectiveness strongly depends on the availability of a large training corpus with an accurate and comprehensive set of annotations, which is usually arduous to create, maintain and extend. On the other hand, dictionary lookup solutions usually only require as input a lexicon consisting in a list of terms within some domain [16–21], for example, a list of names of chemical compounds. The input text is then matched against the terms in the lexicon mainly using string matching techniques. A comprehensive lexicon is normally much easier to find or to create and update than a training corpus, however, dictionary lookup solutions are generally less effective than machine learning solutions.

Searching, filtering and recognizing relevant information in the vast amount of literature being published is an almost daily task for researches working in Life and Health Sciences [22]. Most of them use web tools, such as PubMed [23], but many times to perform repetitive tasks that could be automatized. However, these repetitive tasks are sometimes sporadic and highly specific, depending on the project the researcher is currently working on. Therefore, in these cases, researchers are reluctant to spend resources creating a large training corpus or learning how to adapt highly complex text mining systems. They are not interested in getting the most accurate solution, just one good enough tool that they can use, understand and adapt with minimal effort. Dictionary lookup solutions are normally less complex than machine learning solutions, and a specialized lexicon is usually easier to find than an appropriate training corpus. Moreover, dictionary lookup solutions are still competitive when the problem is limited to a set of well-known entities. For these reasons, dictionary lookup solutions are usually the appropriate option when good enough is what the user requires.

This manuscript proposes a novel dictionary lookup solution, dubbed as minimal named-entity recognizer (MER), which was designed with flexibility, autonomy, and efficiency in mind. MER only requires as input a lexicon in the form of a text file, in which each line contains a term representing a named-entity to recognize. If the user also wants to perform entity linking, a text file containing the terms and their respective Unique Resource Identifiers (URIs) can also be given as input. Therefore, adding a new lexicon to MER could not be easier than this. MER also accepts as input an ontology in Web Ontology Language (OWL) format, which it converts to a lexicon.

MER is not only minimal in terms of the input but also in its implementation, which was reduced to a minimal set of components and software dependencies. MER is then composed of just two components, one to process the lexicon (offline) and another to produce the annotations (online). Both were implemented as a Unix shell script [24], mainly for two reasons: (1) efficiency, due to its direct access to high-performance text and file processing tools, such as grep and awk, and a novel inverted recognition technique; and (2) portability, since terminal applications that execute Unix shell scripts are nowadays available in most computers using Linux, macOS or Windows operating systems. MER was tested using the Bourne-Again shell (bash) [25] since it is the most widely available. However, we expect MER to work in other Unix shells with minimal or even without any modifications.

We deployed MER in a cloud infrastructure to work as an annotation server and participate in the Technical Interoperability and Performance of annotation Servers (TIPS) task of BioCreative V.5 [26]. This participation allowed us to assess the flexibility, autonomy, and efficiency of MER in a realistic scenario. Our annotation server responded to the maximum number of requests (319k documents) and generated the second highest number of total predictions (7130k annotations), with an average of 2.9 seconds per request.

To analyze the statistical accuracy of MER’s results we compared it against a popular dictionary lookup solution, the Bioportal annotator [27], using a Human Phenotype Ontology (HPO) gold-standard corpus [28]. MER obtained the highest precision in both NER and NEL tasks, the highest recall in NER, and a lower processing time. Additionally, we compared MER with Aho-corasick [29], a well-known string search algorithm. MER obtained a lower processing time and higher evaluation scores on the same corpus.

MER is publicly available in a GitHub repository [30], along with the code used to run the comparisons to other systems.

The repository contains a small tutorial to help the user start using the program and test it. The remainder of this article will detail the components of MER, and how it was incorporated in the annotation server. We end by analyzing and discussing the evaluation results and present future directions.

**Fig. 1:**
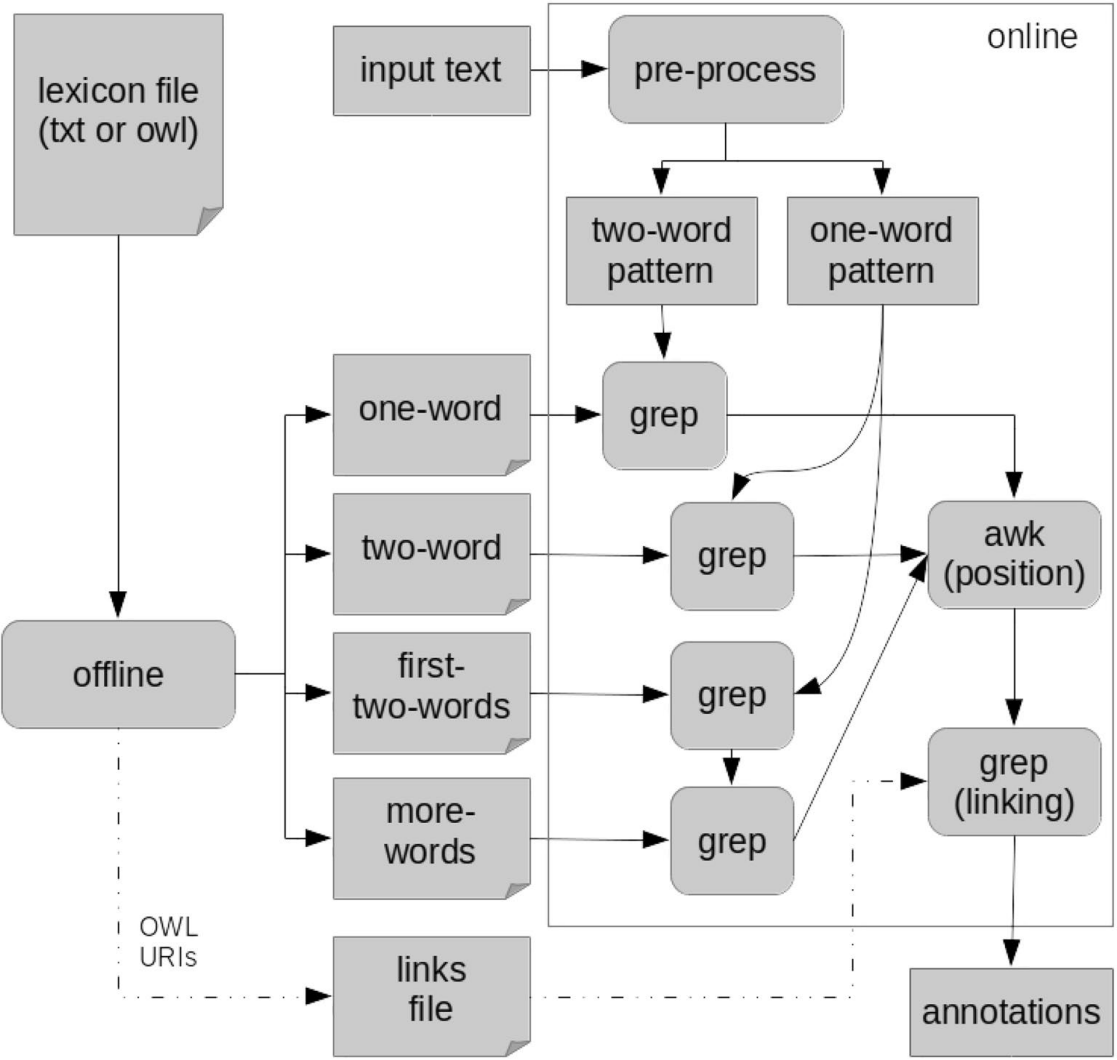
Workflow of MER depicting its inverted recognition technique

## MER

Figure 1 shows the generic workflow of MER. The offline and online modules were implemented in two shell script files, namely *produce_data_files.sh* and *get_entities.sh*, respectively. Both scripts are available in the GitHub repository. The remainder of this section will explain their methodology in detail.

**Fig. 2:**
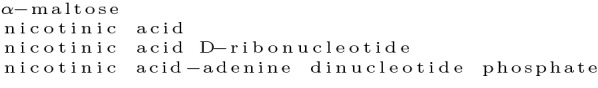
Example of the contents of a lexicon file representing four compounds

**Fig. 3:**
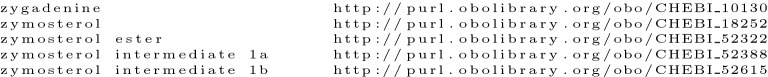
A snippet of the contents of the links file generated with ChEBI

**Fig. 4:**
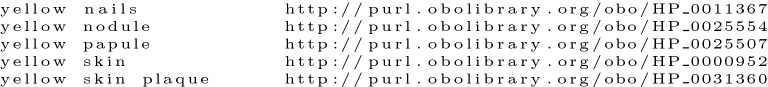
A snippet of the contents of the links file generated with the Human Phenotype Ontology

**Fig. 5:**
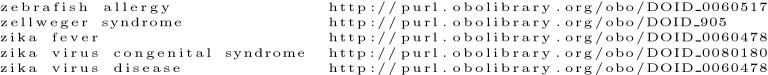
A snippet of the contents of the links file generated with the Disease Ontology

**Fig. 6:**
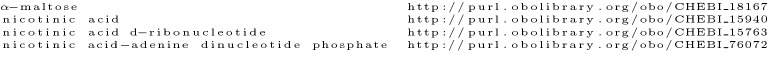
Example of the contents of the links file representing compounds CHEBI:18167, CHEBI:15940, CHEBI:15763 and CHEBI:76072

### Input

Before being able to annotate any text, MER requires as input a lexicon containing the list of terms to match. The user can provide the lexicon as text file (.txt) where each line represents a term to be recognized. Additionally, to perform NEL a tab-separated values file (.tsv) is required. This links file has to contain two data elements per line: the term and the link. Alternatively, the user can provide an ontology (.owl) and MER will automatically parse it to create the lexicon and links files. So if, for example, we want to recognize terms that are present in ChEBI [31], the user can provide the whole ontology (*chebi.owl*) or just collect the relevant labels and store them in a text file, one label per line. Figure 2 presents an example where four ChEBI compounds are represented by a list of terms based on their ChEBI’s name.

**Fig. 7:**
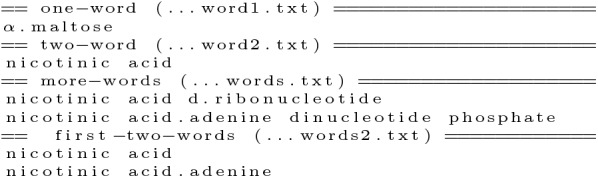
Each block represents the content of each of the four files created after pre-processing the input file shown in Fig. 2

If the user provides an ontology, MER starts by retrieving all the values of the tags *rdfs:label*, *oboInOwl:hasRelatedSynonym* and *oboInOwl:hasExactSynonym* inside each top-level *owl:Class*. The values are then stored in two files: a regular lexicon with a label (term) per line; and a tab-separated values file with a pair term and respective identifier (URI) per line. The links file is then sorted and will be used by MER to perform NEL. Figures 3, 4 and 5 show a snippet of the links files generated for ChEBI ontology [32], HPO [33, 34], and Human Disease Ontology (DOID) [35, 36], respectively.

The links file can also be created manually for a specific lexicon not generated from an ontology. Figure 6 presents the links file created for the lexicon file of Fig. 2.

### Inverted recognition

To recognize the terms, a standard solution would be to apply grep directly to the input text. However, the execution time is proportional to the size of the lexicon, since each term of the lexicon will correspond to an independent pattern to match. To optimize the execution time, we developed the inverted recognition technique. The inverted recognition uses the words in the processed input text as patterns to be matched against the lexicon file. Since the number of words in the input text is much smaller than the number of terms in the lexicon, grep has much fewer patterns to match. For example, finding the pattern *nicotinic acid* in the two-word chemical lexicon created for TIPS is more than 100 times faster than using the standard solution.

To perform the inverted recognition technique, MER splits the lexicon into three files containing the terms composed by one (one-word), two (two-word) and three or more words (more-words). The second step creates a fourth file containing the first two words (first-two-words) of all the terms in the more-words file. During the above steps, MER makes the following minor modifications to the terms: convert all text to lowercase; contiguous white spaces are replaced by one white space; full stops are removed; leading and trailing white spaces are removed; and all special characters are replaced by a full stop. Since some special characters may cause matching problems, MER assumes that all the special characters (characters that are not alphanumeric or a whitespace, for example, hyphens) can be matched by any other character, so these characters are replaced by a full stop, like in regular expressions. Figure 7 presents the contents of each of the four files created using the terms shown in Fig. 2. Note that the word *acid-adenine* was replaced by *acid.adenine*, and the last file presents the first two words of each entry in the third file. Note also that all the above steps are performed offline and only once per lexicon.

**Fig. 8:**
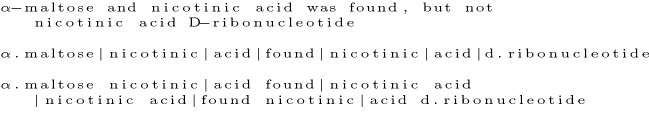
Example of a given sentence to be annotated (first line), and its one-word and two-word patterns created by MER

**Fig. 9:**
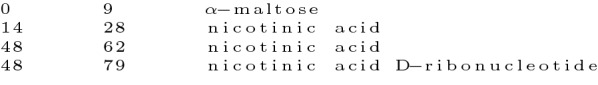
Output example of MER for the sentence in Fig. 8 and the lexicon in Fig. 2 without any links file

**Fig. 10:**
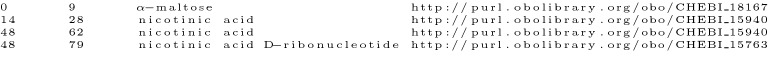
Output example of MER for the sentence in Fig. 8, the lexicon in Fig. 2, and the links file of Fig. 6

**Fig. 11:**
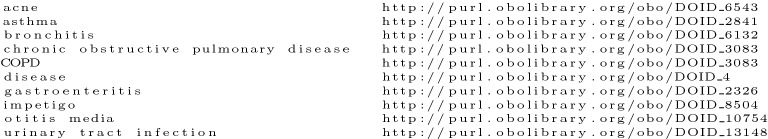
Output example of MER for the abstracts with PubMed identifiers: 29490421 and 29490060, and the Human Disease Ontology

The online module of MER starts when the user provides a new input text to be annotated with a lexicon already pre-processed. The goal is to identify which terms of the lexicon are mentioned in the text. The first step of MER is to apply the same minor modifications to the input text as described above, but also remove stop-words, and words with less than a given number of characters. The file with the list of stop-words and the minimum entity length are parameters that the user can easily modify in the scripts. The list of stop-words used in this study are in the *stopwords.txt* file of the GitHub repository. For this study, we selected 3 as the minimum entity length because two-character acronyms are not so common, and we empirically found that most of the two-character matches were errors.

This will result in a processed input text derived from the original one. Note that MER only recognizes direct matches, if lexical variations of the terms are needed, then they have to be added in the lexicon, for example by using a stemming algorithm. MER will then create two alternation patterns: (1) one-word pattern, with all the words in the input text; and (2) two-word pattern, with all the consecutive pairs of words in the input text. Figure 8 shows an example of these two patterns.

Next, MER creates three background jobs to match the terms composed of: (1) one word, (2) two words, and (3) three or more words. The one-word job uses the one-word pattern to find matching terms in the one-word file. Similarly, for the two-word job, that uses the two-word pattern and file. The last job uses the two-word pattern to find matches in the two-first-word file, and the resulting matches are then used as a pattern to find terms in the more-words file. The last job is less efficient since it executes grep twice, however, the resulting list of matches with the two-first-word file is usually small, so the second execution is negligible. In the end, each job will create a list of matching terms that are mentioned in the input text.

Since the processed input text cannot be used to find the exact position of the term, MER uses the list of matching terms to find their exact position in the original input text. MER uses awk to find the multiple instances of each term in the original input text. The awk tool has the advantage of working well with UTF-8 characters that use more than one byte, in opposition to grep that just counts the bytes to find the position of a match. MER provides partial overlaps, i.e. a shorter term may occur at the same position as a longer one, but not full overlapping matches (same term in the same position). We also developed a test suite to refactor the algorithm with more confidence that nothing is being done incorrectly. The test suite is available in the GitHub repository branch dedicated to development [37].

Figure 9 shows the output of MER when using as input text the sentence in Fig. 8, and the lexicon of Fig. 2. Note that *nicotinic acid* appears twice at position 14 and 65, as expected, without affecting the match of *nicotinic acid D-ribonucleotide*.

### Linking

If the links file is provided, then MER will try to find the recognized term in that file. This step is basically a grep at the beginning of each line in the file, and only returns the first exact match of each term. Figure 10 shows the output of MER when using the links file of Fig. 6 that was missing in Fig. 9. Figure 11 shows the output of MER for two abstracts using the Human Disease Ontology. Note that this functionality was implemented after our TIPS participation [38].

## Annotation server

TIPS is a novel task in BioCreative aiming at the evaluation of the performance of NER web servers, based on reliability and performance metrics. The entities to be recognized in TIPS were not restricted to a particular domain.

The web servers had to respond to single document annotation requests. The servers had to be able to retrieve the text from documents in the patent server, the abstract server and PubMed, without using any kind of cache for the text or for the annotations. The annotations had to be provided in, at least, one of the following formats: BeCalm JSON, BeCalm TSV, BioC XML or BioC JSON.

**Fig. 12:**
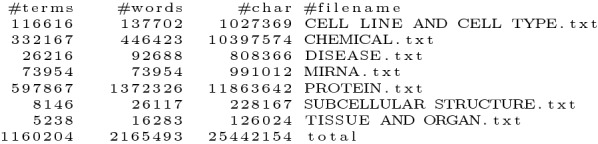
Number of terms, words, and characters in the lexicons used in TIPS, obtained by using the following shell command: wc -lmw *.txt

### Lexicons

The first step to participate in TIPS was to select the data sources from which we could collect terms related with the following accepted categories: Cell line and cell type: Cellosaurus [39]; Chemical: HMDB [40], ChEBI [32] and ChEMBL [41]; Disease: Human Disease Ontology [35]; miRNA: miRBase [42]; Protein: Protein Ontology [43]; Subcellular structure: cellular component aspect of Gene Ontology [44]; Tissue and organ: tissue and organ subsets of UBERON [45].

A post-extraction processing was applied to these data files, which consisted in lowercasing all terms, deleting leading and trailing white spaces and removing repeated terms. Since repeated annotations of different types were not allowed, we created another lexicon containing terms that appeared on more than one of the other lexicons. The terms matched to this lexicon were categorized as Unknown, as suggested by the organization. The software to extract the list of terms from the above data sources can be found in the GitHub repository branch dedicated to TIPS [37].

Figure 12 shows the number of terms, number of words, and number of characters of each lexicon created. Our Annotation Server was then able to recognize more than 1M terms composed of more than 2M words and more than 25M characters. All lexicons are available for reuse as a zip file in the TIPS branch of our repository [37].

**Fig. 13:**
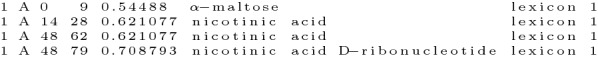
Output example of MER using BeCalm TSV format for the sentence in Fig. 8 and the lexicon in Fig. 2

### Input and output

We adapted MER to provide the annotations in the BeCalm TSV format. Thus, besides the input text and the lexicon, MER had also to receive the document identifier and the section as input. In Fig. 13, the document identifier is 1 and section is A. The score column is calculated by $1-1/\ln (nc)$, where *nc* represents the number of characters of the recognized term. This assumes that longer terms are less ambiguous, and in that case, the match should have a higher confidence score. Note that MER only recognizes terms with three or more characters, so the minimum score is 0.08976 and the score is always lower than 1.

We used jq [46] a command-line JSON processor to parse the requests. The retrieval of each document was implemented using the popular curl tool, and we developed a specific parser for each data source to extract the text to be annotated. The parsers are also available at the TIPS branch [37].

### Infrastructure

Our annotation server was deployed in a cloud infrastructure composed of three Virtual Machines (VM). Each VM had 8 GB of RAM and 4 Intel Core CPUs at 1.7 GHz, using CentOS Linux release 7.3.1611 as the operating system. We selected one VM (primary) to process the requests, distribute the jobs, and execute MER. The other two VMs (secondary) just execute MER. We installed the NGINX HTTP server running CGI scripts given its high performance when compared with other web servers [47]. We also used the Task Spooler [48] tool to manage and distribute within the VMs the jobs to be processed.

The server is configured to receive the REST API requests defined in the BeCalm API documentation. Each request is distributed to one of the three VMs according to the least-connected method of NGINX. When a *getAnnotations* request is received, the server first downloads the documents from the respective sources and then processes the title and abstract of each document in the same VM. Two jobs are spawned in background, corresponding to the title and abstract. Each annotation server handles all the entity types mentioned in Fig. 12, spawning a separate job for each entity type. The name of the entity type is added as another column to the output of Fig. 9. These jobs can run in parallel since they are independent from each other and the output of each job can be easily merged into a final TSV output file. When a job finishes processing, a script checks if the other jobs associated with the same requests have also finished processing. If that is the case, then the results of every job are concatenated and sent back to BeCalm using the *saveAnnotations* method.

**Fig. 14:**
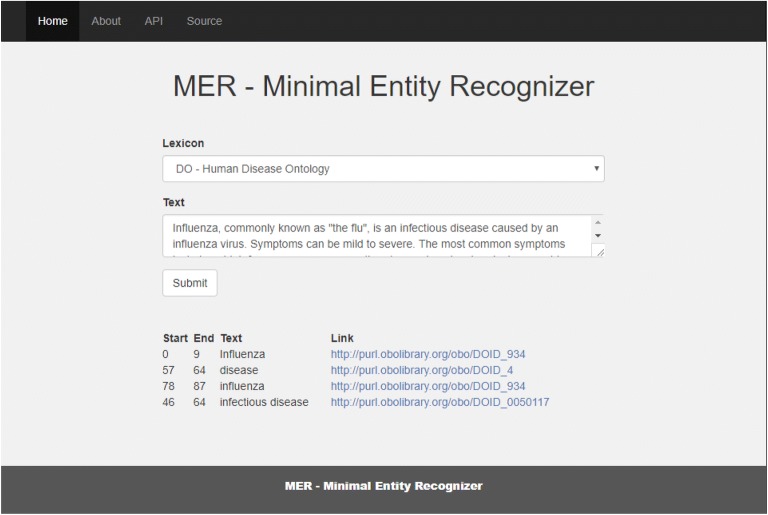
Screenshot of the MER web graphical user interface

To test MER outside of the scope of the TIPS competition, we implemented a different REST API which accepts as input raw text and the name of a lexicon. This way, the document does not have to be retrieved from external sources, and we can evaluate the performance of MER independently. This alternative API can be accessed online, along with a simple user interface shown in Fig. 14 [49].

## Results and discussion

### Computational performance

**Table 1:** Official evaluation results of the TIPS task (time values are in seconds)

	MER	Best
# Requests	3.19E+05	3.19E+05
# Predictions	7.13E+06	2.74E+07
Mean time seek annotations (MTSA)	1.29E−01 s	1.37E−02 s
Mean time per document volume (MTDV)	2.38E−03 bytes/s	8.58E−04 bytes/s
Mean annotations per document (MAD)	2.25E+01	1.01E+02
Average response time (ART)	2.90E+00 s	1.07E+00 s
Mean time between failures (MTBF)	4.58E+06 s	4.58E+06 s
Mean time to repair (MTTR)	0.00E+00 s	0.00E+00 s

Table 1 shows the official TIPS evaluation data of our system [50]. These results refer to the whole period of the competition, from February 5, 2017 to March 30, 2017. The evaluation process and metrics used are described in the workshop article [26]. Each request consisted of one document that the server had to retrieve either from PubMed or a repository hosted by the organization. Our server was able to handle all 319k requests received during the evaluation period, generating a total of 7.13M annotations (second best) with an average of 22.5 predictions per document (MAD) (third best). In average, each prediction has been generated in 0.129 s (MTSA). Our average processing time value (ART) was 2.9 s, and the processing time per document volume (MTDV) was 0.00238 bytes/s. The Mean time between failures (MTBF) and Mean time to repair (MTTR) metrics were associated with the reliability of server, and our team obtained the maximum scores on those metrics.

MER was able to efficiently process the documents by taking less than 3 s on average without using any type of cache. We note that all documents, irrespectively of the source, were annotated using all the entity types presented in the previous Lexicons section. Furthermore, the time to process each document is affected by external sources used to retrieve the document text. If the text is provided with the request, then the processing time should be considerably shorter. Another factor is the latency between our server and the TIPS server. As we were not able to measure this latency, it is difficult to measure the impact on the response times, and it was not taken into consideration for the evaluation metrics.

We compared the time necessary to process the same sentence on the same hardware using MER and a more complex machine learning system, IBEnt [11], using the sentence of Fig. 8. While IBEnt took 8.25 s to process the sentence, MER took only 0.098 s. Although IBEnt is optimized for batch processing, therefore reducing the time per document as the number of documents increases, MER is still 84 times faster than IBEnt in this experiment. Thus, besides being easy to install and configure, MER is also a highly efficient and scalable NER and NEL tool.

Part of the optimization of MER is due to the four files that are generated by the offline module. These files are generated from the lexicon file, which contains one entity per line. For NEL, there is another necessary step, which consists in converting an OWL file in a lexicon. This process took around 15 min for each ontology. However, processing a lexicon file is quite faster, taking 0.746 and 3.671 s for the HPO and ChEBI ontologies, respectively.

### Precision and recall

**Table 2:** Comparison between MER and BioPortal on the HPO gold-standard corpus

	NER			NER+NEL			ART	MTSA
	P	R	F	P	R	F		
BioPortal	0.6862	0.4463	0.5408	0.6118	0.3979	0.4822	1.15E+00 s	1.45E−01 s
MER	0.7184	0.4514	0.5544	0.6155	0.3868	0.4751	7.32E−01 s	9.59E−02 s

We compared the performance of MER with the BioPortal annotator, which is a popular dictionary lookup NER solution. To perform this comparison, we adapted our server to directly receive as input free text, instead of requiring another request to retrieve the documents. We used the HPO corpus to compare the two tools. This corpus is composed by 228 scientific abstracts annotated with human phenotypes, associated with the HPO. We used an updated version of this corpus, which aimed at improving the consistency of the annotations [51]. A total of 2773 textual named entities were annotated in this corpus, corresponding to 2170 unique entity mentions. We compared the quality of the results produced by each tool using the standard precision, recall and F1-score measures, as well as the time necessary to process each document on average (ART) and time per annotation (MTSA).

Table 2 shows the results of this comparison, where NER refers to matching the offsets of the automatic annotations with the gold standard, and NEL refers to matching the URI annotated automatically with the gold standard. As expected, combining both tasks (NER+NEL) results in lower scores than performing only NER. Using MER, the F1-score obtained was 0.5544, while BioPortal obtained an F1-score of 0.5408 on the NER task. Considering the NEL task too, BioPortal obtained a better F1-score than MER, indicating that some entities were linked to incorrect URIs. Bioportal annotator employs a semantic expansion technique that could lead to more accurate URIs, using the relations defined in the ontology [52]. An approach to improve the results would be to incorporate semantic similarity measures, so MER could also consider related classes in the NEL task [53].

However, MER obtained lower response times than BioPortal, in terms of time per document and per annotation. To account for the difference in latency between the two servers, we used the ping tool to calculate the round-trip time of each server, averaged over 10 packets. MER obtained a round-trip time of 6.72E−03 s while BioPortal obtained 1.86E−01 s, representing a difference of 1.79E−01 s. This means that MER had a better connection to the machine we used to run the experiments, but this had minimal impact when comparing to a difference of 4.18E−01 s in both response times (ART).

**Table 3:** Comparison between MER and Aho-corasick on the HPO gold-standard corpus

	NER			ART (s)	MTSA (s)
	P	R	F		
Aho-corasick	0.2282	0.2665	0.2459	0.8596	0.0786
MER	0.7184	0.4514	0.5544	0.5088	0.0667

We also compared MER with a well-known string search algorithm, Aho-corasick using the HPO corpus [29]. In this experiment, we did not attempt to match entities to ontology concepts as this would require additional enhancements to the Aho-corasick algorithm. We used the same HPO lexicon for both methods, as well as the same documents. Unlike the comparison to BioPortal, the experiment was done using local installations of MER and of the Makefast tool [54], which provides an implementation of the Aho-corasick algorithm. Table 3 shows the results of this comparison. MER obtained higher precision, recall and F1-score, as well as a lower processing time per document and per annotation. MER obtained better evaluation scores since it was developed specifically for NER, while Aho-corasick is a generic string search algorithm. The processing time was also shorter, due to the lexicon pre-processing done by the offline module of MER. However, this pre-processing is quick (3.671 s for the HPO ontology) and only has to be done once.

## Conclusions

We presented MER, a minimal named entity recognition and linking tool that was developed with the concepts of flexibility, autonomy, and efficiency in mind. MER is flexible since it can be extended with any lexicon composed of a simple list of terms and its identifiers (if available). MER is autonomous since it only requires a Unix shell with awk and grep command-line tools, which are nowadays available in all mainstream operating systems. MER is efficient since it takes advantage of the high-performance capacity of grep as a file pattern matcher, and by proposing a novel inverted recognition technique.

MER was integrated in an annotation server deployed in a cloud infrastructure for participating in the TIPS task of BioCreative V.5. Our server was fully developed in-house with minimal software dependencies and is open-source. Without using any kind of cache, our server was able to process each document in less than 3 s on average. Performance and quality results show that MER is competitive with state-of-the-art dictionary lookup solutions.

## Abbreviations

NER: named entity recognition
NEL: named entity linking
URI: unique resource identifier
OWL: Web Ontology Language
CHEBI: chemical entities with biological interest
HPO: Human Phenotype Ontology
TIPS: Technical Interoperability and Performance of annotation Servers
MTSA: mean time seek annotations
MTDV: mean time per document volume
MAD: mean annotations per document
ART: average response time
MTBF: mean time between failures
MTTR: mean time to repair
VM: Virtual Machine
